# Test-retest resting-state fMRI in healthy elderly persons with a family history of Alzheimer’s disease

**DOI:** 10.1038/sdata.2015.43

**Published:** 2015-10-13

**Authors:** Pierre Orban, Cécile Madjar, Mélissa Savard, Christian Dansereau, Angela Tam, Samir Das, Alan C. Evans, Pedro Rosa-Neto, John C.S. Breitner, Pierre Bellec, The PREVENT-AD Research Group, Paul Aisen, Tharick Ali Pascoal, Elena Anthal, Melissa Appleby, Alan Barkun, Thomas Beaudry, Pierre Bellec, Fatiha Benbouhoud, Veronique Bohbot, Jason Brandt, John C. S. Breitner, Céline Brunelle, Leopoldina Carmo, Laksanaun Cheewakriengkrai, Louis Collins, Blandine Courcot, Doris Couture, Suzanne Craft, A. Claudio Cuello, Mahsa Dadar, Christian Dansereau, Samir Das, Marina Dauar-Tedeschi, Dorothy Dea, Clément Debacker, René Desautels, Nicole Desrochers, Sylvie Dubuc, Guerda Duclair, Marianne Dufour, Mark Eisenberg, Rana El-Khoury, Pierre Etienne, Alan C. Evans, Anne Marie Faubert, Fabiola Ferdinand, Vladimir S. Fonov, David Fontaine, Renaud Francoeur, Josée Frappier, Joanne Frenette, Serge Gauthier, Valérie Gervais, Renuka Giles, Renee Gordon, Rick Hoge, Bradley T. Hyman, Yasser Iturria-Medina, Clifford R. Jack, Justin Kat, Zaven S. Khachaturian, Stephanie Kliegman, David S. Knopman, Penelope Kostopoulos, Anne Labonté, Marie-Elyse Lafaille-Magnan, Tanya Lee, Claude Lepage, Ilana Leppert, Jeannie-Marie Leoutsakos, Cécile Madjar, Laura Mahar, Jean-Robert Maltais, Axel Mathieu, Sulantha Mathotaarachchi, Gerhard Maultaup, Ginette Mayrand, Diane Michaud, Justin Miron, Thomas J. Montine, John C. Morris, Lisa-Marie Münter, Vasavan Nair, Jamie Near, Holly Newbold-Fox, Pierre Orban, Véronique Pagé, Mirela Petkova, Cynthia Picard, Galina Pogossova, Isabelle Poirier, Judes Poirier, Jens Pruessner, Natasha Rajah, Pierre Rioux, Pedro Rosa-Neto, Mark A. Sager, Mélissa Savard, Reisa A. Sperling, Angela Tam, Pierre N. Tariot, Eduard Teigner, Louise Théroux, Ronald G. Thomas, Paule-Joanne Toussaint, Jennifer Tremblay-Mercier, Miranda Tuwaig, Isabelle Vallée, Vinod Venogopalan, Karen Wan, Seqian Wang

**Affiliations:** 1 StoP-AD Centre, Centre for Studies on Prevention of Alzheimer’s disease, 6875 LaSalle Boulevard, Montreal, QC H4H 1R3, Canada; 2 Centre de recherche de l’Institut Universitaire de Gériatrie de Montréal, 4545 Queen Mary, Montreal, QC H3W 1W5, Canada; 3 Université de Montréal, 2900 Boulevard Edouard-Montpetit, Montreal, QC H3T 1J4, Canada; 4 Douglas Mental Health University Institute Research Centre, 6875 LaSalle Boulevard, Montreal, QC H4H 1R3, Canada; 5 McGill University, 845 Sherbrooke W, Montreal, QC H3A 0G4, Canada; 6 McConnell Brain Imaging Center, Montreal Neurological Institute, 3801 University, Montreal, QC H3A 2B4, Canada; 7 McGill University Research Centre for Studies in Aging, 6825 LaSalle Boulevard, Montreal, QC H4H 1R3, Canada; 8 University of Southern California's Alzheimer's Therapeutic Research Institute, San Diego, CA, USA; 9 John Hopkins University, Baltimore, MD, USA; 10 Wake Forest School of medicine, Winston-Salem, NC, USA; 11 Massachussets Alzheimer’s Disease Research Center, Harvard Medical School, Boston, MA, USA; 12 Mayo Clinic, Rochester, MN, USA; 13 Khachaturian & Associates Inc, Potomac, MD, USA; 14 Washington University, Seattle, WA, USA; 15 Washington University School of Medecine in St-Louis, MO, USA; 16 Wisconsin Alzheimer's Institute, UW School of Medicine and Public Health, Milwaukee, WI, USA; 17 Center for Alzheimer’s Research and Treatment Harvard Medical School, Boston, MA, USA; 18 Banner Alzheimer Institute, Phoenix, AZ, USA; 19 University California, San Diego, School of medicine, La Jolla, CA, USA

**Keywords:** Predictive markers, Alzheimer's disease, Brain imaging, Functional magnetic resonance imaging

## Abstract

We present a test-retest dataset of resting-state fMRI data obtained in 80 cognitively normal elderly volunteers enrolled in the “Pre-symptomatic Evaluation of Novel or Experimental Treatments for Alzheimer's Disease” (PREVENT-AD) Cohort. Subjects with a family history of Alzheimer's disease in first-degree relatives were recruited as part of an on-going double blind randomized clinical trial of Naproxen or placebo. Two pairs of scans were acquired ~3 months apart, allowing the assessment of both intra- and inter-session reliability, with the possible caveat of treatment effects as a source of inter-session variation. Using the NeuroImaging Analysis Kit (NIAK), we report on the standard quality of co-registration and motion parameters of the data, and assess their validity based on the spatial distribution of seed-based connectivity maps as well as intra- and inter-session reliability metrics in the default-mode network. This resource, released publicly as sample UM1 of the Consortium for Reliability and Reproducibility (CoRR), will benefit future studies focusing on the preclinical period preceding the appearance of dementia in Alzheimer's disease.

## Background & Summary

The dementia that results from neurodegeneration in Alzheimer's disease (AD) is associated with disruptions in functional brain connectivity^1,2^. These alterations in brain connectivity can be explored *in vivo* in humans using resting-state functional magnetic resonance imaging (rsfMRI), a technique that captures spontaneous brain function^3^. The detection of spatially distributed networks of temporal synchronization defines so-called resting-state networks^4^. Since the seminal work by Greicius and colleagues^5^, research has confirmed that patients who suffer from dementia of AD exhibit connectivity abnormalities, particularly in the default-mode network (DMN)^6–13^. This network includes brain regions that are consistently more active at rest than during a broad range of tasks^14^. Interestingly, the spatial extent of the DMN overlaps well with regions showing high levels of beta-amyloid deposition in demented patients in AD^15^. Regions of the DMN include the posterior cingulate cortex (PCC), the precuneus (PCUN), the temporo-parietal junction (TPJ) and the anterior cingulate/medial prefrontal cortex (ACC/aMPFC)^16^. Other brain structures are also regarded as part of different subnetworks of the DMN, for instance the medial temporal lobe (MTL) or the superior frontal gyrus (SFG)^17,18^.

Dementia of AD is thought to be the end-stage of a chronic illness where silent, yet critical, neural changes develop well before the appearance of clinical symptoms^2^. At a prodromal stage of AD, patients with mild cognitive impairment (MCI) also exhibit alterations in DMN functional connectivity, as evidenced by rsfMRI^19–22^. Although their daily living is not affected, MCI patients exhibit mild cognitive deficits that are reminiscent of those seen more severely in patients with a dementia of AD and they are at a higher risk of progressing to dementia. Interestingly, cognitively normal elderly (CNE) persons may also show abnormal functional connectivity in similar brain networks in relation to various factors that predispose them to develop AD. For instance, a high beta-amyloid burden or the presence of the Alipoprotein E (APOE) allele 4 are both associated with aberrant functional connectivity of the DMN in elderly persons who do not show any cognitive deficit^23–26^. Altogether, these findings emphasize the need to initiate therapies well before neurodegeneration has gone beyond repair. In order to identify individuals who could benefit from such early therapies, it is thus necessary to identify biomarkers that are predictive of AD many years before the appearance of clinical symptoms. In this context, the study of first-degree relatives of elderly persons suffering from dementia of AD is of a particular relevance. Indeed, AD family history (i.e., sibling or parent) has been shown to impact DMN functional connectivity in rsfMRI, even in APOE 4 allele noncarriers, thus indicating additional genetic risk factors^27^.

A critical prerequisite for the identification of biomarkers based on rsfMRI is to determine the reliability of the functional connectivity patterns evidenced with this technique^28^. To date, test-retest studies looking at the reliability of resting-state networks have been conducted in both young^4,29^ and elderly^30^ healthy subjects. Recent results have revealed that resting-state networks in MCI patients have a decreased reliability^31^ but no studies have yet characterized how a family history of AD might affect the reliability of functional brain connectivity at rest. Notably, not all studies allow differentiating between short-term (within minutes) and long-term (months later) reliability^31^. In addition, these test-retest studies rely on relatively small samples, which preclude drawing strong conclusions from their findings. Here, we report on a rsfMRI dataset obtained in 80 CNE with a family history of AD. Both short- and long-term test-retest data are made freely available as sample UM1 of the Consortium for Reliability and Reproducibility (CoRR), which is described in the overview manuscript by Zuo *et al.*^32^.

## Methods

### Participants

The 80 elderly volunteers (65.4±6.2 years old, 58/22 females/males) are part of the Pre-symptomatic Evaluation of Novel or Experimental Treatments for Alzheimer's Disease (PREVENT-AD) Cohort assembled at the StoP-AD Centre, Montreal, Canada. Participants had to meet the following criteria: (1) family history of parent or multiple degree relative(s) with AD; (2) age 60 or older (age 55 or older if subject is within 15 years of parent's symptom onset); (3) no diagnosable cognitive disorder; (4) good general health to suggest ability to participate in longitudinal studies for >5 years; and (5) for specific biomarker-endpoint trials, no medical condition and/or use of medication that would make it inadvisable for the participant to be assigned to study treatment. Nested within the PREVENT-AD cohort is a subpopulation of individuals who are enrolled in a biomarker-endpoint randomized controlled trial of naproxen, the Naproxen Trial Cohort. The intervention consists of a double-blind randomized trial of 220 mg of Naproxen Sodium or placebo twice daily. Due to the nature of this study, it is impossible to determine for which subjects the retest rsfMRI session at 3 months might have been impacted by medical treatment. Subject-by-subject phenotypic information is provided in Table 1 (available online only), and the ISA-tab metadata associated with this article. All subjects had given informed consent and the study was approved by the "Research, Ethics and Compliance Committee" of McGill University.

### MRI acquisition

Brain imaging data were collected on a 3 T MRI scanner (Magnetom Tim Trio, Siemens; see ISA-tab metadata for details of each scan). Two sessions conducted 111.4±24.3 days apart each included two consecutive runs of 150 functional volumes (each run lasting 5 min 45 s). Functional T2*-weighted images were obtained using a blood-oxygen-level-dependent (BOLD) sensitive, single-shot echo planar sequence (TR=2000 ms; TE=30 ms; FA=90°; matrix size=64×64; voxel size=4×4×4 mm^3^; 32 slices). Structural T1*-weighted scans were acquired using a GRE sequence (TR=2300 ms; TE=2.98 ms; FA=9°; matrix size=256×256; voxel size=1×1×1 mm^3^; 176 slices). Note that dummy scans were automatically rejected during data acquisition.

### MRI preprocessing

Imaging files in the DICOM (Digital Imaging COmmunications in Medicine) format were converted to Neuroimaging Informatics Technology Initiative (NIfTI) format using dcm2nii from MRIcron (http://www.mccauslandcenter.sc.edu/mricro/mricron). Face information was removed from anatomical images by using the FullAnonymize.sh V1.0b script provided by the FCP/INDI group (http://www.nitrc.org/frs/shownotes.php?release_id=1902). During conversion, forty-eight resting-state functional images were inadequately flipped along the z-axis and were thus reoriented using the FSL tools^33^ (http://fsl.fmrib.ox.ac.uk/fsl/fslwiki/Fslutils).

## Data Records

The data of this resource (Data Citation 1) are freely available through either the COllaborative Informatics and Neuroimaging Suite (COINS) Data Exchange (http://coins.mrn.org/dx)^34^ or the Neuroimaging Informatics Tools and Resources Clearinghouse (NITRC; http://fcon_1000.projgects.nitrc.org/indi/CoRR/html/index.html), each requiring a user account prior to download. Images can be downloaded in three archive files (.tar) at the NITRC website along with a comma separated value file including phenotypic data (UM_1_phenotypic_data.csv). Note the description of the terminology used in the latter file can be downloaded under the "Phenotypic data legend" link at the following webpage: http://fcon_1000.projects.nitrc.org/indi/CoRR/html/_static/downloads.html. The COINS Data Exchange provides a more enhanced data querying tool from which you can use specific criteria to download one's target dataset. Both intra- and inter- sessions test-retest data are available. Each subject directory contains two imaging sessions (session_1 and session_2). Within each session folder, there are one anatomical folder (anat_1) with the anatomical image (anat.nii.gz) and two resting-state folders (rest_1 and rest_2), each including one resting-state acquisition (rest.nii.gz). Phenotypic data include age, gender, handedness, resting-state instructions given to the candidates and the time that elapsed between the two sessions of resting-state acquisitions.

## Technical Validation

### rsfMRI data preprocessing

The datasets were preprocessed and analyzed using the NeuroImaging Analysis Kit version 0.12.17 (NIAK; http://www.nitrc.org/projects/niak/), under CentOS with Octave (http://www.gnu.org/software/octave/) version 3.6.1 and the Minc toolkit (http://www.bic.mni.mcgill.ca/ServicesSoftware/ServicesSoftwareMincToolKit) version 0.3.18. Analyses were executed in parallel on the "Guillimin" supercomputer (http://www.calculquebec.ca/en/resources/compute-servers/guillimin), using the pipeline system for Octave and Matlab (PSOM)^35^.

Each fMRI dataset was corrected for slice timing; a rigid-body motion was then estimated for each time frame, both within and between runs, as well as between one fMRI run and the T1 scan for each subject^36^. The T1 scan was itself non-linearly co-registered to the Montreal Neurological Institute (MNI) ICBM152 stereotaxic symmetric template^37^, using the CIVET pipeline^38^. The rigid-body, fMRI-to-T1 and T1-to-stereotaxic transformations were all combined to resample the fMRI in MNI space at a 3 mm isotropic resolution. To minimize artifacts due to excessive motion, all time frames showing a displacement greater than 0.5 mm were removed^39^. The following nuisance covariates were regressed out from the fMRI time series: slow time drifts (basis of discrete cosines with a 0.01 Hz high-pass cut-off), average signals in conservative masks of the white matter and the lateral ventricles as well as the first principal components (accounting for 95% variance) of the six rigid-body motion parameters and their squares^40^. The fMRI volumes were finally spatially smoothed with a 6 mm isotropic Gaussian blurring kernel. A more detailed description of the pipeline can be found on the NIAK website (http://niak.simexp-lab.org/pipe_preprocessing.html).

### Quality control metrics

**Figure 1:**
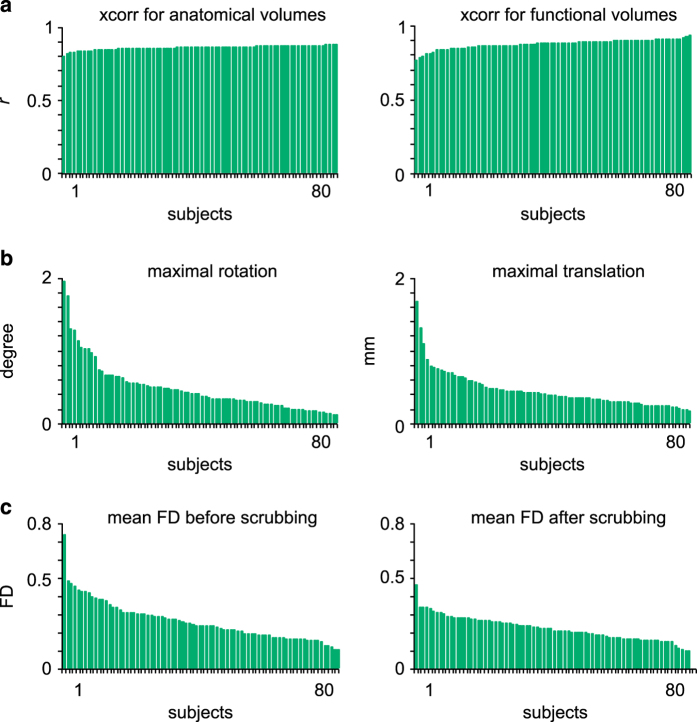
Group-level summary statistics of co-registration procedures and motion parameters. Averages over the two test and two retest runs are shown for all subjects, which are sorted in ascending order in terms of data quality. Spatial correlations between individual anatomical and functional scans are shown for each subject (**a**). Maximal rotation and translation movement parameters are shown for each subject (**b**). The mean frame displacement (FD) values are shown for each subject, before and after scrubbing of volumes with FD>0.5 (**c**). Only runs with >60 volumes left after scrubbing were included in the analyses.

**Figure 2:**
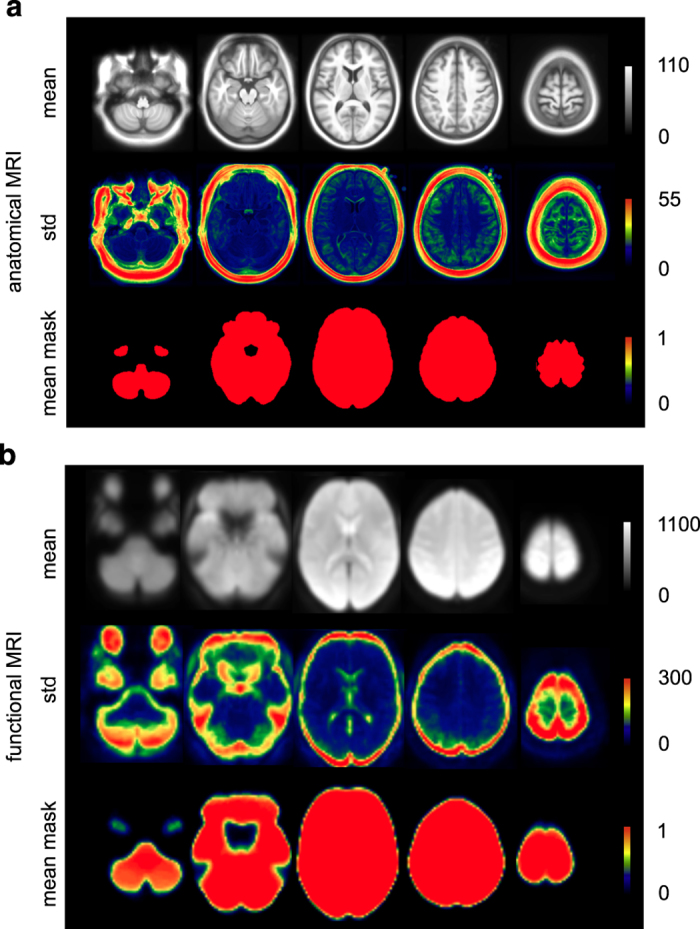
Group-level summary maps of co-registration procedures. Maps (mean across subjects, standard deviation across subjects, mean of the individual brain masks) are shown for both anatomical (**a**) and functional (**b**) MRI.

Group-level summary indices were used for a careful yet rapid quality control of the preprocessing results. These indices included the spatial (Pearson’s) correlation coefficient between the brain volume of each individual and the population average, after non-linear co-registration to stereotaxic space was performed. The co-registration procedures reached satisfactory levels for all subjects (Fig. 1), for both anatomical (r>0.79, 0.85±0.02) and functional (r>0.76, 0.86±0.03) scans. Visual examination of individual datasets further supported this result. A series of summary brain maps were also derived, both for anatomical and functional scans. These maps included the average and standard deviation (std) of all brain volumes after non-linear co-registration, as well as the average of brain masks to identify differences in the field of view across subjects (Fig. 2).

Group-level summary metrics were also generated to determine the maximal transition in motion parameters for each individual (Fig. 1). This allowed us to identify subjects with high levels of motion during the acquisition of the rsfMRI dataset, in terms of maximal transition (0.54±0.31 mm) and rotation (0.47±0.35°). A censoring (scrubbing) method^39^ was used to remove volumes with excessive motion, based on a metric called framewise displacement (FD greater than 0.5). As a consequence, 38 out of 320 runs were rejected from subsequent analyses due to an insufficient number of time points remaining after scrubbing correction (<60 unscrubbed volumes per run, corresponding to 120 s of acquisition with a TR of 2 s). Figure 1 shows the individual FD values for each subject and run, before (raw FD) and after (residual FD) scrubbing. The amount of motion in the PREVENT-AD participants was compared to the motion levels observed in a reference dataset of 25 young healthy adults, the NYU CSC TestRetest dataset^29,41^ (https://www.nitrc.org/projects/nyu_trt/). These reference data were acquired with a TR identical to that of the PREVENT-AD dataset (2000 ms) and include a number of volumes per run in the same range (197 versus 150). The NYU CSC dataset, which passed the quality control procedure with the same criteria as the PREVENT-AD data, was used for comparison with the PREVENT-AD results in all subsequent analyses. The raw and residual FD values averaged across runs were respectively 0.25±0.11 and 0.21±0.07 in the PREVENT-AD sample, leaving 122.05±32.05 usable volumes per run on average across subjects (Fig. 1). Raw and residual FD values significantly differed from those seen in healthy young subjects in the NYU CSC TestRetest dataset (0.2±0.06 and 0.18±0.04, *P*<0.05), in line with previous findings that indicate higher levels of motion in elderly populations^41^.

### Independent selection of candidate brain connections

In order to assess the validity of rsfMRI data in the present PREVENT-AD sample, we aimed to independently identify a series of brain connections that are most strongly impacted in AD, allowing the characterization of point-to-point correlations, i.e., the connectivity between pairs of nodes. To assess test-retest reliability in elderly persons at higher risk of developing AD, it is of the highest clinical relevance to focus the analysis on connections that are specifically known to be affected in AD. To this end, we first performed a literature review and meta-analysis based on a selection of six studies^6–11^ (Table 2). These studies were published prior to 2013, which corresponds to the start of the clinical trial when a priori connections were defined. A test-retest analysis was then conducted on an independent dataset to further narrow down the selection of connections to the most reliable point-to-point correlations.

**Figure 3:**
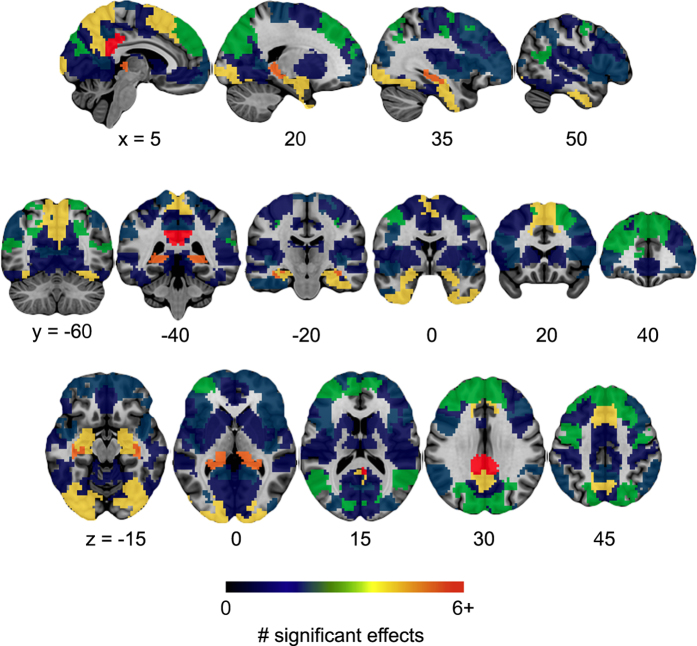
Output map of the meta-analysis. BASC parcels with the highest number of coordinates showing a significant effect across contrasts in the selected studies, indicating the consistency of findings across studies. Maps are superimposed onto the anatomical ICBM 152 template. MNI coordinates are given for sagittal, coronal and axial slices.

There is no single authoritative reference on the effect of AD dementia on rsfMRI connectivity, and the field has been dominated thus far by studies with small samples (n~20) and limited statistical power. Because the DMN has been most extensively studied, we decided to focus on this network and ran a meta-analysis on six papers that (1) used some analog of seed-based connectivity maps in rsfMRI using one or multiple seeds in the DMN; (2) investigated abnormalities in functional connectivity in patients suffering from a dementia of AD; and (3) provided tables of coordinates in stereotaxic space for the results. All the contrasts found in the 6 studies and coordinates of significant effects were entered into a meta-analysis. To assess the degree of consistency across contrasts, we counted the number of coordinates located in each one of the hundred functional parcels obtained from an independent dataset. A data-driven approach, the Bootstrap Analysis of Stable Clusters^43^ (BASC), was used to generate a functional brain parcellation with 100 clusters on the Cambridge sample, which includes ~200 young healthy adults from the 1000 functional connectome database^44^ (https://www.nitrc.org/projects/fcon_1000/). The Cambridge dataset passed the quality control procedure with the same criteria as the PREVENT-AD data. Figure 3 shows the resulting map of the meta-analysis, highlighting the variability across studies, with only a limited number of regions reaching a score above 3 across contrasts. Note that the count of significant effects per cluster is an aggregate of all coordinates, regardless of the fact that they come from one or multiple studies. Table 3 lists the brain regions, and their associated networks, that showed effects reported as significant in the published studies.

Considering that we pooled studies of the DMN, we decided to select all connections of parcels within the DMN, as well as connections between a parcel inside the DMN and a parcel outside the DMN, as candidates. This fairly conservative literature review identified 9 nodes in the DMN (36 point-to-point correlations within the DMN) and 9 seeds in networks outside of the DMN (81 point-to-point correlations between a node in the DMN and a node outside of the DMN). A test-retest analysis based on the NYU CSC TestRetest dataset further narrowed down this selection of 117 candidate connections to 11 measures. In this dataset, each subject was scanned for three rsfMRI runs: two runs separated by 45 min from one another in a single session and another run acquired 5–16 months later. One intra-class correlation (ICC) was generated intra-session, and two ICCs were generated inter-session for all 117 connections. Connections were ranked based on averages of intra- and inter-session ICCs and described based on a standard definition of reliability measures^45^. Our findings were consistent with results previously reported for young adults using the same dataset^29,42^, with a mean ICC over all connections of 0.36 and 30 connections scoring a moderate-to-substantial level of ICC (>0.5). The 11 connections with the highest ICCs (>0.5) were selected for each node and subsequently used in the analyses on the PREVENT-AD sample. Seven connections were retained within the DMN while 4 of them were found between the DMN and another network.

### Seed-based functional connectivity

**Figure 4:**
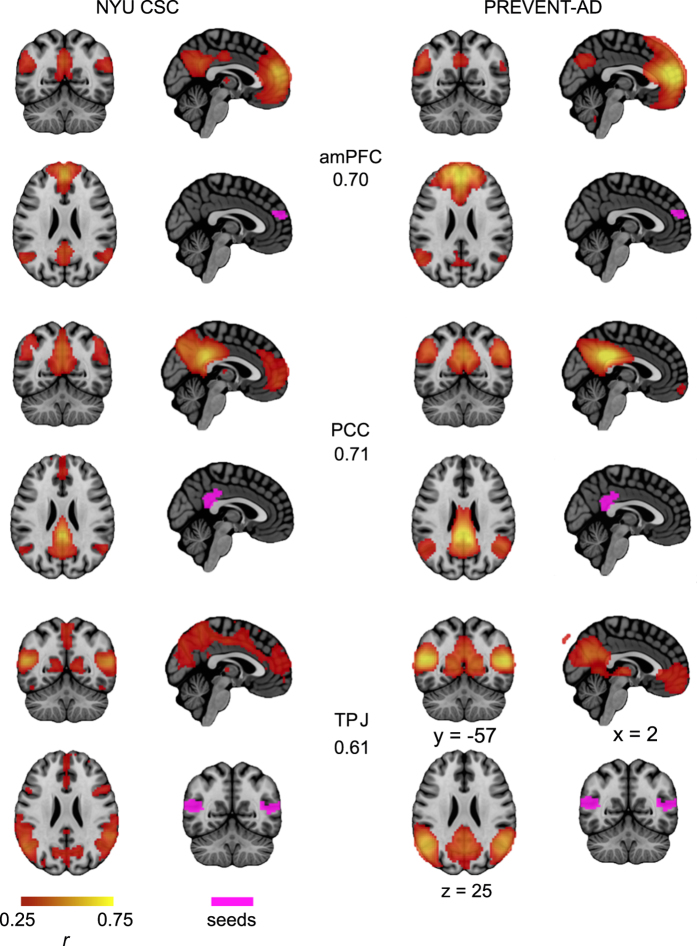
Average connectivity (correlation) maps in the DMN. Maps are shown for 3 seeds located in the anterior medial prefrontal cortex (aMPFC), the posterior cingulate cortex (PCC), and the temporo-parietal junction (TPJ). Correlation values indicate the correspondence for connectivity maps obtained in the NYU CSC and the PREVENT-AD datasets. Maps are superimposed onto the anatomical ICBM 152 template. MNI coordinates are given for representative slices.

To assess the quality of the rsfMRI data in the PREVENT-AD cohort, we extracted average connectivity maps associated with 3 of the seeds in the DMN, as defined by the BASC functional parcels described above: the aMPFC, PCC, and TPJ. Each seed belongs to at least one connection of interest. Connectivity maps were obtained by calculating the Pearson's correlation coefficient between the average time series of the seed region and the time series of all other voxels in the brain. A Fisher transform was further applied on the correlation values to improve the normality of their distribution across subjects. Note that the fMRI data was previously preprocessed and resampled in stereotaxic space, such that an identical seed region could be used for all subjects. For each seed, the average connectivity map of participants in the PREVENT-AD cohort was compared to the average connectivity maps seen in the healthy young subjects of the NYU CSC TestRetest dataset. Visual inspection of the functional connectivity maps suggested a good overlap between the spatial connectivity maps generated in the PREVENT-AD cohort and those seen in young subjects (Fig. 4). DMN maps identified with three different seeds all included typical DMN structures. Pairwise comparisons between functional connectivity maps of the two groups (inclusively masked with the gray matter) indicated a good similarity of connectivity maps across datasets: correlation values were respectively 0.70, 0.71 and 0.61 for the aMPFC, the PCC and the TPJ. The replication of known resting-state structures of the DMN suggest that the functional MRI data in the PREVENT-AD cohort are of standard quality.

### Test-retest reliability

**Figure 5:**
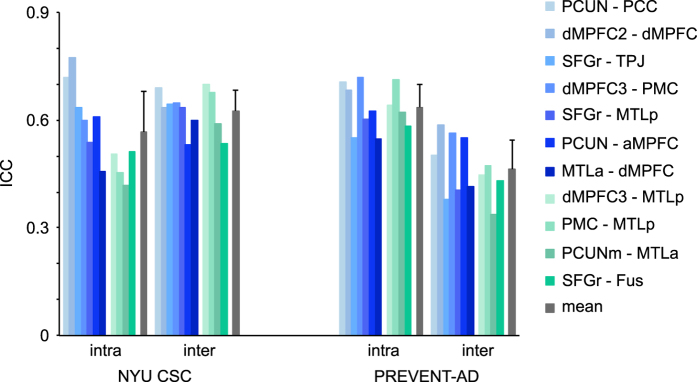
Reliability analysis. Run-averaged reliability was assessed intra- and inter-sessions with intra-class correlation coefficients (ICC). Reliability analyses were performed in the NYU CSC and PREVENT-AD datasets for 11 connections of interest, 7 within the DMN (blue) and 4 between the DMN and other networks (green). See Table 3 for abbreviations.

The quality of the rsfMRI data in the PREVENT-AD cohort was further assessed with a test-retest analysis. Intra- and inter-session ICC values, averaged over runs and connections, were respectively 0.64±0.06 and 0.46±0.08 (Fig. 5). ICC values were in the moderate-to-substantial range, as observed for the healthy young subjects of the NYU CSC TestRetest dataset, where the intra- and inter-session ICC values, averaged over runs and connections, were respectively 0.56±0.11 and 0.63±0.06. An ANOVA performed on z-transformed correlation coefficients indicates a significant interaction effect (*P*<0.005) between the dataset (NYU CSC versus PREVENT-AD) and type of ICC (intra- vs inter-session) variables, with a noticeable smaller inter-session reliability in the PREVENT-AD group. It is worth considering that this measure of reproducibility may be impacted by a potential treatment effect in the elderly population of the PREVENT-AD cohort. We should also note that a lower intra-session ICC in the NYU CSC dataset might be accounted for by a longer delay between runs (45 min versus 0 min in the PREVENT-AD dataset).

Despite such differences, the levels of ICC in the moderate-to-substantial range for the PREVENT-AD dataset were satisfactorily high, consistent with the reliability of rsfMRI previously reported in healthy young subjects^29^, elderly persons with no specific family history of AD^30,31^ and MCI patients^31^. Notably, the sample size of the PREVENT-AD cohort, with 80 subjects, compared favorably to the small sample sizes of such previous studies (~20 subjects per group). The described dataset also has the advantage that both intra- and inter-session can be investigated concomitantly. However, it should be emphasized here that between-session reliability might be confounded by potential drug administration between the test and retest sessions in some subjects and should therefore be considered with caution. It is nonetheless notable that session 2 can be used along session 1 to assess intra-run reliability as both sessions included two runs. In summary, the findings reported here altogether support a standard quality of the rsfMRI data in the PREVENT-AD cohort. This open resource, released publicly as sample UM1 of the Consortium for Reliability and Reproducibility (CoRR)^32^, will expectedly benefit future studies focusing on preclinical AD.

## Additional Information

Table 1 is only available in the online version of this paper.

**How to cite this article:** Orban, P. *et al.* Test-retest resting-state fMRI in healthy elderly persons with a family history of Alzheimer’s disease. *Sci. Data* 2:150043 doi: 10.1038/sdata.2015.43 (2015).

## Supplementary Material



## Figures and Tables

**Table 1:** Phenotypic characteristics

**Subject**	**Age**	**Sex**	**Handedness**
26007	75	female	right
26008	64	female	right
26009	69	female	right
26010	62	female	right
26011	62	female	right
26012	60	female	right
26013	67	male	right
26014	66	female	right
26015	74	female	right
26016	57	female	ambidextrous
26120	62	female	left
26121	66	female	right
26122	65	male	right
26123	58	female	right
26124	65	female	right
26125	63	male	right
26126	65	male	left
26127	83	female	right
26128	64	female	right
26129	71	female	right
26130	79	male	right
26131	73	female	right
26132	67	female	right
26133	61	male	right
26134	72	female	right
26135	62	female	right
26136	71	female	right
26137	61	female	right
26138	62	female	right
26139	67	female	right
26140	58	female	right
26141	58	female	right
26142	62	male	right
26143	77	female	right
26144	72	female	right
26145	61	male	right
26146	62	male	right
26147	60	female	right
26148	61	male	right
26149	61	female	left
26150	82	male	right
26151	64	female	right
26152	70	female	right
26153	73	male	right
26154	62	female	right
26155	84	female	right
26156	63	female	right
26157	75	female	right
26158	61	female	right
26159	61	female	right
26160	63	female	right
26161	61	male	ambidextrous
26162	61	female	right
26163	67	female	right
26164	63	female	right
26165	58	male	right
26166	60	female	right
26167	67	male	right
26168	64	female	right
26169	66	male	right
26170	62	female	right
26171	73	female	right
26172	57	female	right
26173	62	female	right
26174	59	male	right
26175	58	female	right
26176	71	male	right
26177	65	female	right
26178	64	female	right
26179	72	male	right
26180	55	female	right
26181	71	female	right
26182	61	female	right
26183	61	female	right
26184	70	female	right
26185	70	male	right
26186	64	male	right
26187	60	female	right
26188	62	female	right
26189	62	male	right
Basic information is provided for the 80 subjects and their IDs.			

**Table 2:** Literature survey

**Authors**	**Sample**	**Description**
Zhang *et al.*^6^	16 ECN/13 (mild) DAT	Seed-based functional connectivity maps in PCC.
Zhang *et al.*^7^	16 ECN/46 (mild, moderate, severe) DAT	Seed-based functional connectivity maps in PCC.
Wang *et al.*^9^	14 ECN/14 (mild) DAT	Seed-based functional connectivity maps in hippocampus.
Wang *et al.*^8^	14 ECN/14 (very mild) DAT	Seed-based functional connectivity maps in PCC and full brain point-to-point correlations based on an AAL parcellation.
Goveas *et al.*^10^	18 ECN/14 DAT	Seed-based functional connectivity maps in hippocampus.
Damoiseaux *et al.*^11^	18 ECN/21 DAT	Dual-regression independent component analysis (anterior, ventral and posterior DMN components).
List of the references and characteristics of the 6 studies that met the criteria for the meta-analysis.		

**Table 3:** Meta-analysis

**Network**	**Region**	**Label**
Default-mode	posterior cingulate cortex	PCC
	dorsomedial prefrontal cortex	dMPFC
	dorsomedial prefrontal cortex	dMPFC2
	anterior medial prefrontal cortex	aMPFC
	temporo-parietal junction	TPJ
	medial temporal lobe (anterior)	MTLa
	medial temporal lobe (posterior)	MTLp
	precuneus	PCUN
	superior frontal gyrus (right)	SFGr
Visual	fusiform gyrus	FUS
	lingual gyrus	LING
	dorsomedial occipital	DMO
Dorsal attentional	precuneus (motor)	PCUNm
	intra-parietal sulcus	IPS
Sensorimotor	premotor cortex	PMC
Fronto-parietal	dorsolateral prefrontal cortex (left)	DLPFCl
Cingulo-opercular	dorsomedial prefrontal cortex	dMPFC3
Non-applicable	temporal pole	TPo
The brain regions that showed significant effects across studies are listed with their labels and associated networks.		
